# Selections and Abstracts

**Published:** 1913-01

**Authors:** 


### SELECTIONS AND ABSTRACTS

### FEBRUARY MEETING FEDERATION OF STATE MEDICAL BOARD

The Federation of the State Medical Board will hold its annual meeting at the Congress Hotel, Chicago, on Tuesday, February 25th, 1913.

Essayists, eminently qualified, will prepare papers upon the following subjects:

"Is the Universal Reciprocity to be Desired?"

"Should medical boards Require one or more years of college work preliminary to the study of medicine?"

'Should one or more years in a hospital be required for admission to the examination for medical licensure?"

"Rules and regulations governing examinations for medical licensure."

"Qualifications of examiners."

"What fee should be required for the examination?"

"Benefit of having a single federation of state medical boards and method of state board record keeping."

"Means of keeping politics out of state board affairs."

These topics are all of practical and vital interest to medical colleges, medical examining boards, the profession at large and the public.

Those contributing the papers on these subjects come with years of experience and no medical board can afford not to be represented. An earnest and cordial invitation to this meeting is extended to all members of state medical examining and licensing boards, teachers in medical schools, colleges and universities, delegates to the council on medical education of the A. M. A., to the association of American medical colloges and to all others interested in securing the best results in medical education and legislation.

The officers of the federatino are Arthur B. Brown, M. D., President, New Orleans; George H. Hatson, M. D., Secretary-Treasurer, Columbus (State House) Ohio; James A. Duncan, M. D., Chairman Executive Committee, Toledo.

---

### THE GENERAL TREATMENT OF HIGH BLOOD-PRESSURE

By Spencer L. Dawes, M. M., Albany, N. Y.

The general treatment of high blood-pressure would naturally include the treatment by manual methods, regulated movements, and neutral immersion baths, by the means of electricity, and by sodium nitrite; but as papers on each of these particular methods appear in the Symposium and will presumably discuss the methods in detail, they will not be considered under this head. General treatment may be divided into: (1) prophylactic, (2) curative, and (3) symptomatic.

### (1) PROPHYLACTIC TREATMENT

A regulation of the habits of the individual as to *diet, exercise* and *excretion* constitutes the prophylactic measures indicated. Many authorities have arrived at the conclusion that while regulation of the component parts of the daily ration is of value, a far more important factor is in the limitation of the amount consumed. For certainly we must all agree that the best preserved, the most healthy, as well as the least senile of our patients, are those who are temperate in their habits of eating and drinking. Nevertheless it is a fact that a non-nitrogenous diet, with a preponderance of fruits and vegetables, is desirable; while a certain limited number of observers prohibit milk, eggs, cheese, and other articles of diet which are rich in lime salts, and others are insistent that the limitations of the intake of proteins is a factor which should be considered of importance.

Modern rational, and above all, systematic *exercise* will do more to prevent sclerosis of the arteries, and its accompanying hypertension, even than a regulation of the diet. In addition to this, most active American business men should obtain at intervals, certainly once each year, the relaxation attendant upon a sea-voyage or a period in the actual country, away from the excitement of business and society. The digestive apparatus should be kept in the best possible condition and constipation avoided or, if the latter be present, overcome rather by a regulation of the diet than the administration of drugs.

### (2) CURATIVE TREATMENT

Here we should pay our first attention to causation, such as gout, syphilis, the excessive use of alcohol, and overindulgence of all kinds; for, after all, we must admit that high blood-pressure is rather a symptom than a disease.

By far the most popular of all drugs with the idea of cure is *iodine* in some form, given oftentimes to the point of toleration. While potassium iodide is probably most frequently used in this country, the greater number of the French and German clinicians prefer sodium iodide, believing it to be better borne by the digestive tract; and, latterly, many so-called organic preparations of iodine have been introduced and highly recommended by the pharmaceutical houses which are sponsors for their birth.

Whichever of the salts of iodine is used, however, it is usually given in the form of a saturated solution, starting with 2 drops (which, of course, represent about 2 grains of the drug) three times each day, and each day the size of the individual dose is increased by 1 drop until 15 drops are reached, provided the stomach does not rebel. This dosage is then continued with occasional intervals of omission, a week or so at a time, for several years.

Balfour's method in the treatment of aneurism with attendant high blood-pressure he states to be as follows: "So soon as the average pulse rate in recumbency is accurately ascertained, 2 grains of potassium iodide are given every eight hours, and if the pulse rate remains unchanged the dose of iodide may be increased up to 15 grains or more every eight hours, raising it by 1 grain each dose until the pulse rate begins to rise. It is only rarely that we can increase the dose beyond 10 grains without this taking place. When the pulse rate begins to rise the iodide is stopped for one or two days, and then we go back to the highest dose that did not raise the pulse rate and continue the dose."

This use of iodine is often attended by unpleasant digestive symptoms, as well as other evidences of toxemia, and its value rests more upon empirical evidence than upon accurate measurement of the blood-pressure with a manometer. Its use is almost as strongly deprecated as it is highly commended. It is most desirable, in the use of iodine, to recollect that it should not be administered during the period of digestion, because not only is it theoretically a fact that iodine and starch produce an insoluble iodide of starch, but there are the disagreeable effects of iodine upon the digestive tract, too well known to need description here.

(3) Symptomatic Treatment

Unfortunately most of our efforts must be directed toward relief, for the majority of our patients do not come to us until a distinct arteriosclerosis is established, and, as we have already seen, it is more often than not impossible to benefit them by curative treatment.

One of the most ancient methods in the treatment of high blood-pressure, as well as the most popular, was *venesection*, abandoned because of abuses attendant upon its use. Nevertheless, in appropriate cases it often gives brilliant results. Says Wilks: "You see your patient sitting up in bed, the face calm and lips blue or purple, and the jugular veins starting out of the neck and often visibly pulsating, the heart beating quickly and perhaps a tricuspid bruit, indicating the gorged right heart and obstructed lungs; the veins in the body are full to bursting." And here, as well as in uremia, eclampsia, and certain cases of cerebral hemorrhage, emphysema, and pulmonary engorgement, the removal of 10 to 20 ounces of blood will prove a most satisfactory remedial measure.

Results are gratifying to the physician as they are pleasing to the patient often attend upon the free use of *calomel* or the mass of mercury, followed by a brisk *saline purge*. These measures give relief not only by the withdrawal of fluid from the intestinal viscera, with a consequent lowering of arterial tension, but by the removal from the intestines of toxic material which, by its absorption into the circulation, increases the blood-pressure; to this, I am sure, each one of us can testify.

Where there is intestinal torper, with decomposition of the animal food, the use of the lactic-acid tablets (*Bacillus bulgaricus*) may be employed with happy results, overcoming not only digestive disturbances and the accompanying borborygmus, but the secondary production of hypertension.

The promotion of free diuresis in certain types of cases where there is an inefficiency of the skin and kidneys and myocardial degeneration, with or without valvular disease, is often of great help. Here *digitalis* is of greatest value, notwithstanding the fact that primarily it increases the arterial tension, because of its effect on the heart as well as upon the secreting functions of the kidney. For the same reason *theobromine sodiosalicylate is* often helpful; and while the effects of this drug are, in the main, produced through an increased secretion by the kidneys, yet it has a direct effect upon the vasomotor center in the medulla, and thus, secondary to this, upon the blood-pressure. A cobination of these two remedies has, at times, in my experience, given results which, to the patient at least, seemed almost miraculous.

For immediate effect upon hypertension, as well as for continued use over a long period, no class of drugs equals the *nitrites*, and of these, that most frequently employed is what is generally, although improperly spoken of as nitroglycerin. Unfortunately the effect of the nitrites is evanescent, and the appropriate dose must be repeated at frequent intervals in order to prevent the secondary rise in blood-pressure which usually follows its primary effect. The physiological effect of the nitrites is to relax the arteries through their influence on the vasomotor system, and, as is generally the case with all rapidly acting drugs, the primary effect is not only transient, but is followed by a secondary effect directly the opposite. In addition, if large doses are given over a long period (which is the best way to give these agents), we are in danger of lowering the blood-pressure to a point where the secreting function of the kidneys is unfavorably affected. For rapidity of action in extreme cases, amyl nitrite is the most efficient; but its fleetlng effect, as well as its disagreeable smell, makes it undersirable for continued use. As has been noted, nitroglycerin is most in favor, but many careful observers are strong in their preference for sodium nitrite.[1] Erythrol tetranitrate has lately been much lauded, and it has the advantage of producing a more lasting effect; but it unfortunately is an exceedingly expensive drug for prolonged use, which is just what we need it for. It should be stated that the dosage of nitroglycerine, as well as the frequency of the dose, is many times greater than is commonly supposed. In this connection I cannot help but quote what Le Fevre says: "In the treatment of cardiac and renal diseases, the greatest danger, next to the abuse of digitalis, is the unjustifiable administration of the nitrites. High arterial tension is relatively a compensating condition, and it is to be treated only when it becomes excessive. The action of the nitrites is transient, and the use of large doses at frequent intervals is apt to produce increasing irritability of the eardiovascular system, and the patient is more liable to suffer from dyspeneic attacks. There is also danger of the nitrites reducing the tension temporarily below the point at which the kidney secretion can be carried on." Not all cases where there is high blood-pressure demand or even admit a reduction of the tension, for as long as cardiac power compensates for obstruction in the circulation no special treatment is needed.—*Monthly Cyclopedia and Medical Bulletin, January,* 1913.

---

### EXAMINATION OF DENTISTS FOR THE U. S. ARMY

The Surgeon General of the Army announces that examinations for the appointment of Acting Dental Surgeons will be held at Fort Slocum, New York; Columbus Barracks, Ohio; Jefferson Barracks, Missouri; Fort Logan, Colorado; and Fort McDowell, California, on Monday, April 7, 1913.

Application blanks and full information concerning these examinations can be procured by addressing the "Surgeon General, U. S. Army, Washington, D. C."

The essential requirements to securing an invitation are that the applicant shall be a citizen of the United States, shall be between 21 and 27 years of age, a graduate of a dental school legally authorized to confer the degree of D. D. S., and shall be of good moral character and habits.

Acting Dental Surgeons are employed under three years' contract (at the rate of $150.00 per month. They are entitled to traveling allowances in obeying their first orders, in changing stations, and in returning to their homes at termination of service. They also have the privilege of purchasing certain supplies at the Army Commissary. After three years' service, if found qualified, they are promoted to the grade of dental surgeon with the rank of first lieutenant, and receive thereafter the pay and allowance appertaining to that rank.

In order to perfect all necessary arrangements for examination, applications must be in the possession of the Surgeon General at least two weeks before the date of examination. Early attention is, therefore enjoined upon all intending applicants. There is at present a large number of vacancies to be filled.

